# Behavioral and biomedical factors associated with lifestyle modification practices among diagnosed hypertensive patients in pastoral health facilities of southern Ethiopia

**DOI:** 10.3389/fcvm.2024.1450263

**Published:** 2024-12-24

**Authors:** Tagese Yakob, Begidu Yakob, Mesfin Menza Jaldo, Desalegn Dawit, Chernet Elias, Eskinder Israel, Awoke Abraham

**Affiliations:** ^1^ School of Public Health, College of Health Science and Medicine, Wolaita Sodo University, Sodo, Ethiopia; ^2^ Division of Nutrition, Maternal and Child Health Unit, Wolaita Zone Health Department, Wolaita Sodo, Ethiopia; ^3^ Department of Biostatic and Epidemiology, School of Public Health, College of Health Science and Medicine, Wachemo University, Hossana, Ethiopia; ^4^ Department of Public Health, College of Medicine and Health Sciences, Hawassa University, Hawassa, Ethiopia

**Keywords:** behavioral, biomedical, hypertension, lifestyle modification practice, pastoral

## Abstract

**Background:**

More than 23 million deaths and 36.5% of disability-adjusted life-years are the result of the direct effects of unhealthy behavior alone. Daily behaviors have strong implications for health outcomes and quality of life. The aim of this study is to determine the behavioral and biomedical factors associated with lifestyle modification practices among diagnosed hypertensive patients in pastoral health facilities of southern Ethiopia.

**Methods:**

A facility-based cross-sectional study was conducted among 453 diagnosed hypertensive adult patients in pastoral health of southern Ethiopia from June 1/2023 to July 30/2023. The study population was randomly selected from among patients diagnosed with hypertension that was followed up during the study period using a systematic random sampling technique. The data were entered into Epi-Data-4.6.0.2 and exported to SATAT version 14 for analysis. A binary logistic regression model was fitted to determine independent predictors of lifestyle modification practices among hypertensive patients. An adjusted odds ratio with a 95% confidence interval was used to declare a state of significance.

**Results:**

Out of 453 potential participants approached, 433 agreed to successfully participate in the study, for a response rate of 95.6%. Of the total participants, 56.1% (95% CI, 51.38–60.74) of the patients practiced the recommended lifestyle modifications. Alcohol consumption (AOR = 0.64, 95% CI: 0.42–0.96), ever-practiced reducing salt intake (AOR = 2.48, 95% CI: 1.57–3.93), and low-density lipoprotein cholesterol levels in the blood (>160 mg/dl) (AOR = 3.3, 95% CI: 1.72–6.34) were independently associated with lifestyle modifications in patients with hypertension.

**Conclusion:**

This study revealed that the prevalence of lifestyle modification practices (LMP) was low among hypertensive patients. Lifestyle modification is not one-stop practical, but continuous proper awareness creation, counseling, and health education and health promotion are needed to scale up healthy behavior in patients with hypertension to create a good lifestyle.

## Background

Hypertension is a global public health challenge due to its high prevalence and associated risk of stroke and cardiovascular diseases in adults (1). The World Health Organization (WHO) estimates that more than one billion people worldwide are affected by high blood pressure, with a prevalence of hypertension of one in every three adults (2). According to a recent finding, there were 75 million adults living with HTN in SSA (3, 4). In Ethiopia, research has shown a gradual increase in the total number of HTN cases (5).

The exact causes of high blood pressure are not known, but several risk factors and conditions may play a role in its development (6). Lifestyle modification is a non-pharmacological therapy that is the keystone of the management of hypertension (4). Various health conditions are caused/exacerbated by unhealthy behaviors (7). Lifestyle modification may facilitate drug step-down and drug withdrawal in highly motivated individuals who achieve and maintain lifestyle changes (4).

More than 23 million deaths and 36.5% of disability-adjusted life-years are the result of the direct effects of unhealthy behavior alone (8). Daily behaviors have strong implications for both short- and long-term health outcomes and quality of life (7). Health-enhancing behaviors promote well-being, reduce disease risk, and improve quality of life. It is estimated that approaching a healthy lifestyle progressively extends life expectancy by 14.0 years for female adults and 12.2 years for male adults in the US (9).

In 2019, the Personalized Prevention of Chronic Diseases consortium recommended the identification of biomarkers that could be used for the prevention of chronic diseases (8). According to the 2019 Global Burden of Disease Study, biomarkers such as systolic blood pressure (SBP), elevated fasting plasma glucose (FPG), and high body mass index (BMI) are associated with disease burden (7). A study in 2018 estimated that the disease burden in Australia due to high cholesterol levels contributed to 2.7% and 37% of coronary heart disease cases, respectively (10). Many people are unaware that they have out-of-range levels of blood lipids despite many studies. Among adults in Australia, two in three had above-normal range blood lipid levels, 57% had uncontrolled out-of-range biomarkers, and only 6.6% of adults who were followed up and taking modifying medication had normal biomedical marker levels; the remaining adults were unaware (11). Studies revealed that approximately 50% of hypertensive patients were living a sedentary lifestyle (6).

Pastoralists are people who practice extensive livestock production system as a livelihood (12). Despite making up a sizable section of the rural population in many developing nations, pastoralists typically receive inequitable health services and interventions (13, 14). Due to inadequate communication, logistical needs, ambiguous civil status, remoteness, and a general sense of low priority, nomadic groups are frequently overlooked at the national level (15, 16).

Currently, the number of hypertensive patients is increasing due to different risk factors, such as unhealthy diets and physical inactivity, in the majority of Ethiopia (17, 18). The majority of the study participants were unfamiliar with recommended lifestyle modification practices (LMP) (9, 19). Hypertension is a major public health problem and today, one in three adults has hypertension (4). Many studies have focused on a small number of categories, such as genetic and demographic variables, family history of chronic disease, age, ethnicity and sex, as these variables are not modifiable and have been shown to be associated with outcomes (17–20). This may support timely interventions, equitably, as well as their integration with digital technologies, to maintain the best possible balance in the lifetime health trajectory (20, 21). However, currently, noninvasive, highly specific and sensitive variables, such as biomarkers, have great diagnostic value, simplifying the evaluation and assessment of individuals particular for specific diseases, their prognostic value capabilities, and their responses to therapeutic patterns (22). Therefore, the objective of this study was to assess behavioral and biomedical factors associated with LMP among diagnosed hypertensive patients in pastoral health facilities of southern Ethiopia.

## Methods and materials

### Study setting, design, and period

A facility-based cross-sectional study was conducted from June 1/2023 to July 30/2023 among diagnosed adult hypertensive patients in public hospitals in pastoral zones of Southern Ethiopia. Based on the latest population projection of the Central Statistics Agency of Ethiopia, the population of the study area was projected to be 3,157,673 [with 473 597 urban (15%) and 2,684,076 rural (85%) residents] (23). During the year of this study, there were 8 hospitals in during study period that deliver compressive service. This area in Ethiopia is impacted by intercommunal conflict (24).

### Population

All known hypertensive patients who attended the hospital southern regional state of Ethiopia for medical treatment follow-up were recruited, and the study population was randomly selected from among diagnosed hypertensive patients with respective hospital who underwent follow-up during the study period. Hypertensive patients aged ≥ 18 years who attended medical treatment follow-up 6 months before the inception of the study were included, while patients who were unable to communicate properly were excluded from the study.

### Sample size determination and sampling procedure

The sample size was calculated by using Epi-data 3.1 by considering the extent of the lifestyle modification practice study conducted in Durame and Nigist Eleni hospitals (27.3%) (17). The sample size was determined for exposure status by using variable cases among exposed individuals (54.95%) and cases among unexposed individuals (40%) for good knowledge about the disease, with an AOR of 1.83, and a ratio of exposed to unexposed individuals of 1 was assumed. The sample size was determined by 95% CI, 80% power, and exposure. Therefore, the total sample size of the study was 412, and after adding a nonresponse rate (10% = 41), the final sample size was 453. The same method was used by similar studies (17, 25). The study was conducted at five selected public hospitals. During start, work up of a sampling frame using the patient's medical registration number from each hospital hypertension registration book was prepared. After that, the calculated sample size was proportionally distributed to each hospital. The study participants were then picked from each of the selected hospital using a computer-generated specific random sampling method.

### Study variables

The outcome variable of the study was LMP. The independent variables of the study included socioeconomic and demographic factors, such as age, sex, place of residence, religion, ethnicity, marital status, level of education, occupational status, and socioeconomic status; behavioral factors, such as unhealthy diet, physical activity, salt consumption, cigarette smoking, alcohol consumption, weight management, KAP of patients toward LSM behaviors, household food insecurity status, and nutritional diversity; and biomedical factors and individual health profiles, such as family history of hypertension, comorbidity, total cholesterol level, triglyceride, HDL cholesterol, LDL cholesterol, blood pressure, and fasting blood sugar level.

### Data collection procedures

The quantitative primary data were collected using structured pretest questionnaires via face-to-face interviews in areas where the privacy of the clients was maintained. The questionnaires included questions about socio-demographic and economic factors, behavioral factors, biomedical/clinical factors, and anthropometric measurements.

Lifestyle modification practices were measured using questionnaires adapted from hypertension self-care activity level effects, which are recommended by the Joint National Committee on Detection, Prevention, Evaluation, and Treatment of Hypertension (JNC7), works in the literature, and the WHO stepwise approach for non-communicable disease surveillance by considering the national situations of the study subjects (26). This tool was initially written in English and then translated into the Amharic version. The Amharic version was again translated back to English to check for consistency of meaning by another person. One senior experienced BSc nurse as a supervisor, two experienced senior nurses who were data collectors and two senior laboratory technologists collected 5 ml venous blood samples from each participant who fasted 9–12 h overnight to check the lipid status of patients by using a BS-200 Chemistry Analyzer machine made in SHENZHEN MINDRAY to analyze blood chemistry results. Lipid profiles include high-density lipoprotein cholesterol (HDL), low-density lipoprotein cholesterol (LDL), triglycerides (TG), and fasting blood sugar (FBG) (27).

The principal investigator provided training for 1 day to have enough knowledge of the techniques, ethics of data collection and quality, and completeness of the data collection process. The data collectors and supervisors were assigned to the hospital where the study was conducted, and the data collection process lasted 2 months.

### Data quality control

One-day training was provided for the data collectors as well as a supervisor by the principal investigator to increase awareness of the data collection techniques, ethical considerations, and quality of the data. Before the actual data collection, a pretest was conducted on 5% of a similar population at Bele Primary Hospital. Based on the findings of a pretest, modifications and development of the tool were made. The data collectors were informed to check the completeness of each questionnaire, whether every question had been completely answered, and whether the supervisor rechecked the completeness of the questionnaire immediately after submission.

### Operational and term definitions

Adherence to lifestyle modifications: respondents who were adherent to diet-, exercise-, smoking-, and alcohol consumption-related recommendations (18).

DASH: a diet rich in fruits and vegetables with low sodium, reduced saturation, and total fat. Diet-related adherence: In this study, respondents who reported that they usually or always consumed a diet rich in vegetables, grains, and fruits; rarely or never consumed salt; and rarely or never consumed foods rich in spices and saturated fat were considered adherent (18).

Exercise-related adherence: respondents who reported that they exercised for >30 min per day at least three times per week (18).

Knowledge about hypertension: Respondents with scores above the mean value on the hypertension evaluation of lifestyle and management scale were considered to have good knowledge about hypertension (18).

Good LMP: Participants responded with a mean score of the recommended lifestyle practice questions or above (28).

Poor LMP: Participants who responded with a score below the mean score on recommended lifestyle practice questions (28).

Current alcohol drinkers are those who consumed alcoholic drink within the past 30 days (18). Current smokers are those who smoke tobacco products daily (29).

Lipid profile: Total cholesterol >200 mg/dl, triglyceride >150 mg/dl, HDL-cholesterol (<40 mg/dl for men, <50 mg/dl for women), LDL-C > 100 mg/dl, and FBG ≥ 126 mg/dl (27).

Vigorous-intensity activities are activities that generate large increases in breathing or heart rate for at least 10 min continuously (27).

Moderate-intensity activities are activities that produce small increases in breathing or heart rate for at least 10 min continuously (27).

High servings of fruits and vegetables: more than five servings of fruits and vegetables (29).

Favorable self-care practices included taking medication regularly, monitoring BP ≥2 times/month, engaging in physical activity ≥4 days a week, trying to keep one's weight down, and not smoking (30).

### Data analysis

The data were checked for completeness, coded, and entered into Epi-data version 4.6.0.2. Data cleaning and analysis were performed with STATA version 14, and descriptive statistics, such as frequencies, proportions, means, standard deviations, and tables, were used to present the results of the study. Bivariate and multivariable binary logistic regression analyses were used to determine the associations between different variables, and odds ratios with 95% confidence intervals (CIs) were calculated for the degree of association between dependent and independent variables to identify important determinants of LMP.

Multivariate analysis was performed for variables with *p* values less than or equal to 0.25 in the bivariate analysis to assess possible confounders. Both crude and adjusted odds ratios (AORs) with 95% confidence intervals (CIs) were reported. During the analysis, Hosmer and Lemeshow's test was performed to check model fitness. A *p*-value < 0.05 was considered to indicate statistical significance.

### Ethical statement

This study was approved and waived by the institutional review board (IRB) of the ethical review committee of the College of Health Sciences and Medicine of Wolaita Sodo University. The study was conducted following the relevant guidelines, regulation, and principles of the Helsinki Declaration. Also permission letter to conduct the study was obtained from Wolaita Sodo Comprehensive Specialized Hospital. Additionally, written informed consent was obtained from the study participants before data collection. The confidentiality of the information was maintained by avoiding any personal identifiers, such as the patient's name, on the questionnaires during the data collection. The information obtained from the participants who were used only for the study was kept confidential and did not harm them. Finally, the recorded data were kept safe by locking them in the locker, and the key of the lock was accessed only by the principal investigator.

### Patient and public involvement

No patients or the public were involved in the design, analysis, or interpretation of this study, and they were not involved in the dissemination of the results.

## Results

### Socio-demographic characteristics of the study subjects

Out of the 453 study participants approached, 433 (95.6%) participated successfully. The mean [±Standard deviation (SD)] age of the participants was 47.64 ± 11.31 years. Greater than half of 241 (55.7%) of the participants were males. About 126 (29.1%) of the respondents were farmers, and about 128 (29.6%) had a primary education. Nearly three-fourth 317 (73.2%) of the participants were resided in urban. Greater than two-thirds of the 299 (69.1%) participants were married, and more than half of the 236 (54.5%) participants had a monthly income of 150 ETB or above (Table 1).

**Table 1:** Socio-demographic characteristics of the behavioral and biomedical factors associated with LMP among diagnosed hypertensive patients in pastoral health facilities of southern Ethiopia.

Variables (*n* = 433)	Category	Male (%)	Female (%)
Age in years	18–40 (early adulthood)	71 (30.2)	59 (29.8)
	41–64 (middle adulthood)	126 (53.6)	133 (67.2)
	>65 (late adulthood)	38 (16.2)	6 (3.0)
Occupation	Housewife	0 (0.0)	109 (55.1)
	Government employee	41 (17.4)	24 (12.1)
	Farmer	103 (43.9)	23 (11.7)
	Merchant	41 (17.4)	24 (12.1)
	Other^a^	50 (21.3)	18 (9.1)
Marital status	Single	55 (23.4)	21
	Married	156 (66.4)	143
	Divorced	12 (5.1)	6 (3.0)
	Widowed	12 (5.1)	28 (14.1)
Educational status	No formal education	60 (25.5)	68 (34.3)
	Primary	63 (26.8)	68 (34.5)
	Secondary	77 (32.8)	36 (18.1)
	College and above	35 (14.9)	26 (13.1)
Monthly income (ETB)	500 and Below	66 (28.1)	42 (21.2)
	501–1,500	49 (20.9)	40 (20.2)
	1,501 and Above	120 (51.0)	116 (58.6)
Residents	Rural	175 (74.5)	142 (71.7)
	Urban	60 (25.5)	56 (28.3)

ETB, Ethiopian Birr.

^a^ NGO, daily laborer, pensioned, student.

### Participant-clinical characteristics

Among the participants, more than half of the patients (220; 50.8%) were aged 6 months to 5 years, whereas approximately 171 (39.5%) patients were diagnosed with hypertension (6–10 years). Of the total patients, 189 (43.6%) were overweight, whereas 226 (52.2%) were normal weight. Approximately one-third (36%) of the respondents had comorbidities, especially kidney disease, and 109 (25.2%) had a family history of hypertension (Table 2).

**Table 2:** Participant clinical-related characteristics of behavioral and biomedical factors associated with LMP among diagnosed hypertensive patients in pastoral health facilities of southern Ethiopia.

Variables (*n* = 433)	Category	Male (%)	Female (%)
Time since diagnosed	6 months to 5 years	135 (57.4)	85 (42.9)
	6–10 years	82 (34.9)	89 (44.9)
	11–15 years	18 (7.7)	24 (12.1)
BMI (kg/m^2^)	Normal (18–24)	143 (60.8)	83 (41.9)
	Overweight (24–28)	86 (36.6)	103 (52.0)
	Obese (>28)	6 (2.6)	18 (9.1)
Family history of hypertension	Yes	76 (32.3)	80 (40.4)
	No	159 (76.7)	118 (59.6)
Multiple comorbidity	Yes	66 (228.1)	43 (21.7)
	No	169 (71.9)	155 (78.3)
Multiple comorbidity types (*n* = 109)	Diabetic mellitus	24 (36.3)	12 (27.9)
	Kidney disease	18 (27.4)	25 (58.1)
	Heart failure	24 (36.3)	6 (14.0)

BMI, body mass index.

### Behavioral and biomedical characteristics of the study participants

Among the participants, nearly half (214, 49.4%) had a history of having ever perceived information about hypertension to modify LSM. The majority, 427 (98.615), knew where to obtain information related to LSM practices, and more than half, 241 (55.7%), had a history of currently taking any herbal or traditional remedies for increased blood pressure. Approximately one-third of the 162 (37.41%) patients practiced lifestyle modification practices such as physical activity/exercise, and more than two-thirds of the 299 (69.1%) practiced LSMs such as diet (any item and amount). Overall, 150 (34.6%) patients practiced reducing their salt intake, whereas 126 (29.1%) practiced their BP frequently as measured by a doctor (twice a day). Of the total participants, 195 (45.0%) had ever consumed alcohol, three hundred seventy-eight 92 (21.3%) had ever smoked any tobacco product, and 109 (25.2%) had ever chewed khat.

According to the laboratory profiles of the participants, for the majority of the patients, 192 (44.3%) had optimal (<100 mg/dl) LDL levels in their blood, greater than half of the 234 (54.0%) respondents had low (<40 mg/dl) HDL levels, and greater than one-third of the 169 (39.0%) respondents had normal (40–60 mg/dl) HDL levels in their blood. However, the majority of the respondents, 333 (76.9%), had normal (<200 mg/dl) total cholesterol (TC) levels in their blood (Table 3).

**Table 3:** Behavioral and biomedical factors associated with LMP among diagnosed hypertensive patients in pastoral health facilities of southern Ethiopia.

Variables (*n* = 433)	Category	Frequency	Percent (%)
Ever perceive the information of hypertension to modify our LMP	Yes	214	49.4
	No	219	50.6
Know where to get information related to LMP practice?	Yes	427	98.6
	No	6	1.4
Currently taking any herbal or traditional remedy for your raised blood pressure	Yes	241	55.7
	No	192	44.3
Ever use alcohol consumption	Yes	195	45.0
	No	238	55.0
Ever smoking tobacco product	Yes	92	21.3
	No	341	78.7
Ever chewing khat	Yes	109	25.2
	No	324	74.8
Ever had lifestyle modification practices like physical	Yes	162	37.4
	No	271	62.6
Ever practiced LMP like dietary (any item and amount)	Yes	299	69.0
	No	134	31.0
Ever practiced reducing salt intake	Yes	150	34.6
	No	283	65.4
Ever practiced your BP frequently measured by the doctor (twice a day)	Yes	126	29.1
	No	307	70.9
LDL	<100 mg/dl (optimal)	192	44.3
	100–159 mg/dl (normal)	65	15.0
	≥160 mg/dl (high)	176	40.6
HDL	>60 mg/dl (high)	30	6.9
	40–60 mg/dl (normal)	169	39.0
	<40 mg/dl (low)	234	54.0
TC	<200 mg/dl (normal)	333	76.9
	≥200 mg/dl (high)	100	23.1

LMP, lifestyle modification practice; BP, blood pressure; TC, total cholesterol; LDL, low density lipoprotein; HDL, high density lipoprotein.

### Adherence to LMP among hypertensive patients

The mean (±SD) weight management score of the patients was 9.71 (±1.5), with a maximum score of 15; nearly three-fourths (309, 71.4%) of the study participants practiced recommended health weight management. Of the patients, 291 (67.1%) practiced the recommended low-salt diet, with the mean (±SD) score for the low-salt diet being 12.1 (±1.4). Of the 433 study participants, 31.4% engaged in activities that produced small increases in breathing or heart rate for at least 10 min continuously, but only 26.6% engaged in regular physical exercise for at least 3 days of the week with a minimum duration of 30 min (exercise-related adherence). Only 46.2% of respondents knew about recommended LMP, with a mean score of 14.32 (±1.6). The mean (±SD) score for diet adherence was 12.87 (±2.26), with a maximum score of 21, with only 19 (4.3%) practicing high serving of fruits and vegetables per day of physical activity for 30 min per day. Only 64 (14.4%) of the participants practiced a history of chewing (Table 4).

**Table 4:** Behavioral and biomedical factors associated with LMP among diagnosed hypertensive patients in pastoral health facilities of southern Ethiopia.

Variables (*n* = 433)	Category	Male (%)	Female (%)
Weight management practice	Bad	65 (27.7)	59 (29.8)
	Good	170 (72.3)	139 (70.2)
Diet-related adherence	Poor adhered	211 (89.8)	156 (78.8)
	Good adhered	24 (10.2)	42 (21.2)
Knowledge about lifestyle modification hypertension	Good adhered	67 (28.5)	59 (29.8)
	Bad adhered	168 (71.5)	139 (70.2)
Exercise-related adherence	Adhered	68 (28.9)	47 (23.7)
	Not adhered	167 (71.1)	157 (76.3)
Physical activity intensity adherence	High	75 (31.9)	61 (30.8)
	Low	160 (68.1)	137 (69.2)
Serving fruits and vegetables adherence	High serving	15 (6.4)	4 (2.0)
	Low serving	220 (93.6)	194 (98.0)
Overall LMP	Good	117 (49.8)	126 (63.6)
	Poor	118 (50.2)	74 (36.4)

LMP, lifestyle modification practice.

**Figure 1:**
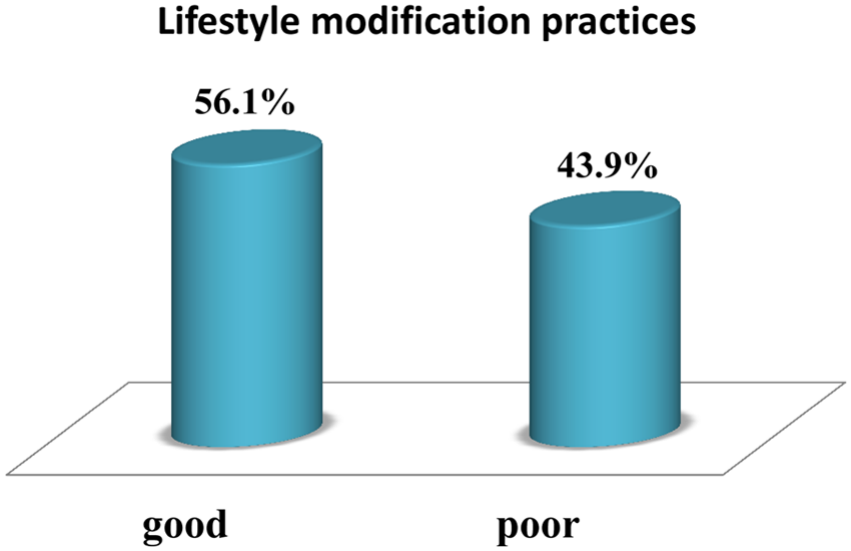
LMP among diagnosed hypertensive patients in pastoral health facilities of southern Ethiopia.

Overall, the mean score for LMP of the participants in this study was 12.99 (SD ± 1.6), with a maximum score of 17. Adherence to LMP among hypertensive patients was 243 (56.1%) (95% CI, 51.38–60.74) (Figure 1). Among adherence to LMP among hypertensive patients them greater than half 117 (48.1%) were male and 126 (51.9) were female and had good lifestyle modification practices for hypertension.

### Factors associated with LMP among hypertensive patients

The results of the bivariate analysis revealed that alcohol consumption, chewing khat, practicing physical activity/sport, ever perceiving the information, currently taking any herbal or traditional remedy, ever practicing reducing salt intake, and low-density lipoprotein were significantly associated with the dependent variables.

After controlling for the possible confounding effects of other covariates, alcohol consumption, ever practiced reducing salt intake, and low-density lipoprotein significantly affected adherence to healthy lifestyle modifications among hypertensive patients. Patients with a history of alcohol consumption were 36% less likely to have good recommended healthy lifestyle modification practices than patients with no history of alcohol consumption (AOR = 0.64, 95% CI: 0.42–0.96). Patients who ever practiced reducing their salt intake were 2.48 times more likely to practice recommended healthy lifestyle modification practices than were patients whose counterparts practiced reducing their salt intake (AOR = 2.48, 95% CI: 1.57–3.93). Additionally, patients whose low-density lipoprotein cholesterol level in the blood was reported (>160 mg/dl) were 3.3 times more likely to practice healthy lifestyle modifications (AOR = 3.3, 95% CI: 1.72–6.34) than those whose blood lipoprotein cholesterol level in the blood was <100 mg/dl (normal) (Table 5).

**Table 5:** Binary and multivariable logistic regression analysis of behavioral and biomedical factors related to LMP among known hypertensive patients in pastoral health facilities of southern Ethiopia.

Variables	Category	LMP (%)		COR (95% CI)	AOR (95% CI)	*p*-value
		Good	Poor			
Ever alcohol consumption	Yes	122 (50.2)	73 (38.4)	0.62 (0.42–0.91)	0.64 (0.42–0.96)	0.035*
	No	121 (49.8)	117 (61.6)	1 (reference)	1 (reference)	
Ever practiced physical activity/sport to control your blood pressure	Yes	82 (33.7)	80 (42.1)	1.43 (0.96–2.11)	1.44 (0.94–2.21)	0.093
	No	161 (66.2)	110 (57.9)	1 (reference)	1 (reference)	
Ever perceive the information of modifying LMP as helpful	Yes	129 (53.1)	85 (44.7)	0.71 (0.48–1.10)	0.71 (0.46–1.07)	0.098
	No	114 (46.9)	105 (55.3)	1 (reference)	1 (reference)	
Currently taking any herbal or traditional remedy for your raised blood pressure	Yes	124 (51.0)	117 (61.6)	1.53 (1.05–0.26)	1.41 (0.93–2.16)	0.101
	No	119 (248.9)	73 (38.4)	1 (reference)	1 (reference)	
Ever chewing khat/tobacco product	Yes	53 (21.8)	56 (29.5)	1.49 (0.97–2.32)	1.57 (0.95–2.49)	0.074
	No	190 (78.8)	134 (70.5)	1 (reference)	1 (reference)	
Ever practiced reducing salt intake to control your blood pressure	Yes	73 (30.0)	77 (40.5)	1.58 (1.10–2.36)	2.48 (1.57–3.93)	<0.001*
	No	170 (70.0)	113 (59.4)	1 (reference)	1 (reference)	
LDL (mg/dl)	<100	127 (52.2)	65 (34.2)	1 (reference)	1 (reference)	
	100–159	41 (16.9)	24 (12.6)	1.14 (0.63–2.10)	1.32 (0.69–2.51)	0.391
	≥160	75 (30.9)	101 (53.2)	2.63 (1.72–4.02)	3.39 (2.10–5.48)	<0.001*

COR, crude odd ratio; AOR, adjusted odd ratio; CI, confidence interval; df, degree of freedom; LMP, lifestyle modification practice; LDL, low density lipoprotein.

* Significant variable at *p* < 0.05 and [Hosmer and Lemeshow test (chi-square = 14.35, df, 8 sig = 0.073)].

## Discussion

Although adopting a healthy lifestyle is one of the most significant ways to manage hypertension, failing to follow through on a healthy lifestyle is one of the main causes of serious complications, uncontrolled hypertension, and the waste of healthcare resources. Therefore, both pharmaceutical and non-pharmacological therapies are needed to control hypertension. Since practicing a healthy lifestyle is the ultimate strategy for controlling hypertension, this study aimed to assess behavioral and biological factors associated with lifestyle modification practices among diagnosed hypertensive patients.

Overall, 56.1% of the participants in this study had good lifestyle practices (95% CI, 51.38–60.74). The results of our study were lower than those of a study conducted in Addis Ababa Yekatite 12 Hospital 46.4% (31) and in Turkey in which 75% of hypertensive patients engaged in recommended lifestyle modifications (32). However, the results of this study align with those of a study conducted in North–Western Nigeria (28), where 56.7% of patients had good adherence and in the Amhara region of the Oromia special zone (52.7%) (33). However, this percentage is greater than that reported in a study conducted in nigeria 16.4% (34), in Bahir Dar City Hospitals, North West Ethiopia 32.4% (35), in Addis Ababa public hospital 23% (3), Dessie 23.6% (18), in Durame (27.3%) (17), and in Central Gondar Zone that was 24.2% (36). This might be due to the increased educational level of patients and the increased level of awareness about lifestyle modification and its advantages. This might also be due to patients relying not only on medication but also on the effects of healthy lifestyle modifications on hypertension control. This might also be due to the similarities of some parts, such as the study design used in all studies.

Patients with a history of alcohol consumption were 36% less likely to have good recommended healthy lifestyle modification practices for hypertension than patients with no history of ever alcohol consumption. This finding aligns with a study from India (4), a study conducted in southeastern Nigeria (37), a school-based study in Berhampur, Odisha (38), and Kedah in Malaysia (39), and a study conducted in northwestern Nigeria (28). A possible explanation may be that patients who do not seek alcohol consumption are more likely to change their lifestyles by not wasting their time on alcohol addiction. In addition, patients with no alcohol-seeking behavior may have a good standard of living and can improve their lifestyle. Currently, health recommendations state that individual daily alcohol consumption limits lead to better health (40) and that individuals should abstain from alcohol intake (41).

Patients who had ever practiced reducing their salt intake were 2.48 times more likely to practice recommended healthy LMP for hypertension than patients who had ever practiced reducing their salt intake (AOR = 2.48, 95% CI: 1.57–3.93). This study supported a study conducted in India that showed that salt restriction had a strong association with LMP in individuals with hypertension (4). Additionally, another study was conducted in North–Western Nigeria (37) and a study conducted on non-pharmacologic therapy among hypertensive patients in Bishoftu, Ethiopia (42). The possible reason might be that adequate information, as explained by mass media, has the power to have an average positive effect size of 5% to promote healthy behavioral change (43). This might also be due to good knowledge about lifestyle modification in hypertensive patients' scale-up and their ability to practice.

According to current study, BMI was not a significantly associated with the good lifestyle modification of hypertensive patients. In contrary to the study conducted in Ambo town, Ethiopia (44), in Tabriz, Iran (45), in Northern china adults (46–48), in USA (49), and in France (50). Further current study also contrary with systemic review and study conduct in cross three populations in Africa and Asia (51, 52). This might be due to lack of continued counseling' and health education current study setting.

LDL levels are significantly associated with lifestyle modifications in hypertensive patients. Patients whose blood LDL levels were high (>160 mg/dl) were 3.3 times more likely to practice healthy lifestyle modifications than those whose blood lipid cholesterol levels were <100 mg/dl (normal). This finding is comparable with that of a study conducted in Saudi Arabia, which showed that significant hypercholesterolemia is a predictor (53). This is because patients with high cholesterol levels have practical hearing and are eager to practice recommended activities, and counseling by healthcare professionals regarding lifestyle modification of biomarker individual profiles is the utmost necessity to ensure a long normal life for hypertensive patients (54). Additionally, improving biomedical-related risk factors has the power to prevent disease, prolong disease progression to improve therapeutic efficacy, and enhance the health of the population (55).

Moreover according to current study, physical activity was not a significant factor influencing the good lifestyle modification of hypertensive patients. This contrary to study conducted in Durame and Nigist Elleni Mohamed Memorial General Hospitals in southern Ethiopia (17), in Addis Ababa, Ethiopia (56), India (57), and in the USA African-Americans (58). This could due to individual awareness level as well as having knowledge about the effect of physical activity on hypertension. Being physically inactive plays a major role in development of hypertension, as well as worsening the condition and physical inactivity causes 9% of premature mortality (59).

Limitations of the study: The main drawback of this study was that it was conducted in public health facilities and did not include home-based follow-up or private follow-up patients. Seasonal variation in access to care and the diagnosis of hypertensive and epidemiology of hypertensive in women with age differences was not consider for current study. Inference of causality is not allowed due to the cross-sectional study design. Additionally, self-reported methods of measurement are related and depend on the participant's memory, and there may be recall bias.

## Conclusion

This study revealed that LMP are rare among hypertensive patients. Healthcare provider should give due attention for patients' history of alcohol consumption, a practice of reducing salt intake and low-density lipoprotein. Lifestyle modification is not one-stop practical, but continuous proper awareness creation, counseling, and health education and health promotion are needed to scale up healthy behavior in patients with hypertension to create a good lifestyle. Further, Healthcare should focus on systems that allow for behavioral change and biomedical profile makeup based on schedules in depth for hypertensive patients in adherence management, as well as a means of accurately assessing adherence to a healthy lifestyle.

## Data Availability

The raw data supporting the conclusions of this article will be made available by the authors, without undue reservation.
